# Development and validation of a scoring system for mortality prediction and application of standardized W statistics to assess the performance of emergency departments

**DOI:** 10.1186/s12873-021-00466-8

**Published:** 2021-06-16

**Authors:** Jinwoo Jeong, Sung Woo Lee, Won Young Kim, Kap Su Han, Su Jin Kim, Hyungoo Kang

**Affiliations:** 1 Department of Emergency Medicine, Dong-A University, College of Medicine, 49201 DaesinGongwon-Ro 26, Seo-Gu, Busan, South Korea; 2 Department of Emergency Medicine, Korea University, College of Medicine, 02841 Goryeodae-Ro 73, Seongbuk-Gu, Seoul, South Korea; 3 Department of Emergency Medicine, University of Ulsan, College of Medicine, Asan Medical Center, 05505 Olympic-Ro 43-Gil 88, Songpa-Gu, Seoul, South Korea; 4 Department of Emergency Medicine, Hanyang University, College of Medicine, 04763 Wangsimni-Ro 222-1, Seongdong-Gu, Seoul, South Korea

**Keywords:** Health care evaluation mechanisms, Health care quality, access, and evaluation, Prognosis, Hospital mortality

## Abstract

**Background:**

In-hospital mortality and short-term mortality are indicators that are commonly used to evaluate the outcome of emergency department (ED) treatment. Although several scoring systems and machine learning-based approaches have been suggested to grade the severity of the condition of ED patients, methods for comparing severity-adjusted mortality in general ED patients between different systems have yet to be developed. The aim of the present study was to develop a scoring system to predict mortality in ED patients using data collected at the initial evaluation and to validate the usefulness of the scoring system for comparing severity-adjusted mortality between institutions with different severity distributions.

**Methods:**

The study was based on the registry of the National Emergency Department Information System, which is maintained by the National Emergency Medical Center of the Republic of Korea. Data from 2016 were used to construct the prediction model, and data from 2017 were used for validation. Logistic regression was used to build the mortality prediction model. Receiver operating characteristic curves were used to evaluate the performance of the prediction model. We calculated the standardized W statistic and its 95% confidence intervals using the newly developed mortality prediction model.

**Results:**

The area under the receiver operating characteristic curve of the developed scoring system for the prediction of mortality was 0.883 (95% confidence interval [CI]: 0.882–0.884). The Ws score calculated from the 2016 dataset was 0.000 (95% CI: − 0.021 – 0.021). The Ws score calculated from the 2017 dataset was 0.049 (95% CI: 0.030–0.069).

**Conclusions:**

The scoring system developed in the present study utilizing the parameters gathered in initial ED evaluations has acceptable performance for the prediction of in-hospital mortality. Standardized W statistics based on this scoring system can be used to compare the performance of an ED with the reference data or with the performance of other institutions.

## Background

To improve the quality of emergency care, performance measurement is essential [1]. Performance measures include structure, process, and outcome indicators [1, 2]. Outcome measures evaluate the effect of patient care and include readmission, mortality, or patient satisfaction [1]. Outcome indicators have many advantages, such as being valid, stable and concrete, so policymakers and patients have shown greater interest in outcome measures [1, 2]. In-hospital mortality or short-term mortality are commonly used outcome indicators to evaluate the outcome of emergency department (ED) treatment [3, 4]. However, crude mortality has limitations, as it is difficult to interpret the results when differences in severity are not considered. Therefore, scoring systems based on physiological variables were developed and have been used to measure the severity of illness or injury [5–7]. The impact of ED process indicators such as ED length of stay or leaving without being seen are evaluated against outcome indicators such as hospital mortality [3, 8]. However, mortality without consideration of severity can lead to misleading or inconclusive results. For example, although Singer et al. found that the length of ED stay was associated with increased mortality, the finding is not conclusive because it is possible that more severely ill patients are likely to stay longer in the ED. [9]

In the field of trauma care, methods of comparing performance between institutions or trauma systems have been developed and widely used, including the W statistic and standardized W (Ws) based on the Trauma and Injury Severity Score (TRISS) [6, 7, 10–14]. Although several scoring systems and machine learning-based approaches have been suggested to grade the severity of illness in ED patients, methods of comparing the severity-adjusted mortality of general ED patients between different systems have yet to be developed [15, 16].

We hypothesized that W statistics used in trauma systems could be used to assess ED performance when coupled with an appropriate scoring system that can predict mortality in general ED patients. The aim of the present study was to develop a scoring system for predicting the mortality of ED patients based on data collected at the initial evaluation and to validate the usefulness of the scoring system for comparing severity-adjusted mortality between institutions with different severity distributions by means of the W and Ws statistics.

## Methods

### Study setting and population

The study was based on the registry of the National Emergency Department Information System (NEDIS) maintained by the National Emergency Medical Center of the Republic of Korea. The NEDIS is a computerized system that prospectively collects demographic and clinical data pertaining to ED patients from all emergency medical facilities in Korea [17, 18]. The Institutional Review Board of the Dong-A University Hospital determined the study to be exempt from the need for informed consent because the study involves a deidentified version of the preexisting national dataset. (DAUHIRB-EXP-20-062).

Data regarding consecutive emergency visits between January 1, 2016, and December 31, 2017, were extracted from the NEDIS registry, anonymized by the National Emergency Medical Center and provided for this research. Data from the designated regional emergency centers or local emergency centers were included for analysis, while cases from smaller EDs were excluded because of excessive missing data. Regional emergency centers are the highest-level emergency centers in Korea and have been designated as such by the Ministry of the Health and Welfare, while local emergency centers are designated by the governors or mayors and are intended to provide local people with access to emergency medical care [18]. Data from 2016 were used for the construction of the prediction model, and data from 2017 were used for validation. Children aged less than 15 years, patients who transferred from the ED, patients who were in cardiac arrest at the time of arrival, and patients with missing data or highly unreliable values (such as blood pressure above 300 mmHg or pulse oximetry above 100%) were excluded from analysis.

### Measurements

Age, sex, cause of visit, level of consciousness measured with the alert, verbal, pain, unresponsive (AVPU) scale, systolic and diastolic blood pressures, heart rate, respiratory rate, body temperature, pulse oxygen saturation at the time of ED arrival, and treatment results at the time of ED discharge and at the time of hospital discharge were retrieved from the NEDIS database. The cause of visit was divided into disease and other.

The outcome variable was defined as mortality, which included mortality in the ED, hospital mortality after admission. Discharge with almost no chance of recovery and the expectation of death in the short term were also regarded as mortality [19, 20].

### Data analyses

#### Building the mortality prediction model

Each measured variable was plotted against mortality, and score values were arbitrarily assigned to ranges of each variable because it could not be assumed that each measurement has a linear association with mortality. Logistic regression was performed with all the variables initially included. Variables with multicollinearity and negative effects were removed when the final model was built. To simplify the prediction system, the final score for each variable was calculated as two times the arbitrary score multiplied by the coefficient derived from the logistic regression, rounded to an integer value. Finally, the scores were summed to generate the mortality prediction score, which is referred to as the ‘Emergency Department Initial Evaluation Score (EDIES)’ in this article.

#### Validation of the mortality prediction score

Receiver operating characteristic (ROC) curves were drawn with the EDIES against mortality with data from 2016 and 2017, and the areas under the curves were calculated.

#### Standardized W statistic for comparing performance between systems

The W statistic is the difference between the actual number of survivors and the predicted number of survivors per 100 patients analyzed. The W statistic has been used to compare the performance of trauma care systems, with predictions based on the trauma and injury severity score (TRISS) [6, 11]. However, the W statistic was criticized for being inadequate to compare systems with different severity distributions; therefore, the standardized W (Ws) statistic was proposed, where the predicted probability of survival is divided into a number of intervals, and the W statistic is calculated for each interval [13]. We calculated the Ws statistic and its 95% confidence intervals in the same manner as described in the literature [13], except that EDIES was used for prediction instead of the TRISS.

#### Validation of the Ws statistics

**Fig. 1:**
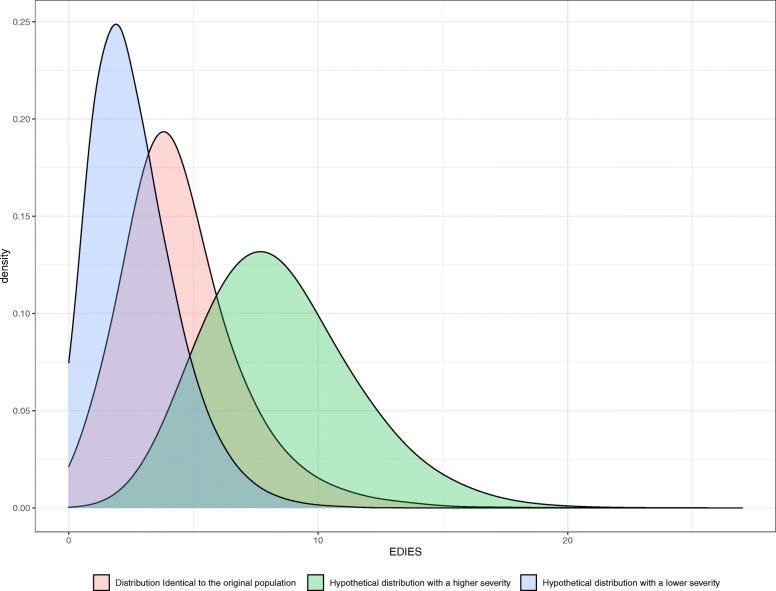
Hypothetical distribution of severity scores identical to the original population, nonsevere, and severe samples. The model distributions were used to validate the stability of the standardized W statistics from samples of different severity case distributions compared with the original validation population

To evaluate whether the Ws statistics and their 95% confidence intervals can be used to compare performance between systems with different severity distributions, random, severe, and not severe cases were sampled from the 2017 database. Hypothetical severe cases were sampled according to the gamma distribution with a shape parameter of 9 and a rate parameter of 1, while hypothetical nonsevere cases were sampled with a shape parameter of 3.4 and a rate parameter of 1.1. The parameters were determined based on trial-and-error to reflect extremely high or low levels of severity. (Fig. 1) For each severity category, 30,000 cases were sampled repeatedly 1000 times each. Actual Type I error rates of the calculated 95% intervals of the Ws statistic were calculated by the percentage of samples for which the population Ws statistic from the entire 2017 data was outside of the 95% confidence intervals of the sample Ws statistic [21, 22].

#### Statistical software used

MedCalc Statistical Software version 19.0.7 (MedCalc Software Ltd., Ostend, Belgium) was used to determine the correlation of factors with univariate mortality and curve fitting with linear or quadratic functions.

R version 3.6.1 (R Foundation for Statistical Computing, Vienna, Austria, 2019) was used for the other statistical analyses. The package ‘stats’ was used for logistic regression and sampling cases matched to different severity distributions. The package ‘pROC’ was used for receiver operating curve analysis.

## Results

### Characteristics of study subjects

**Fig. 2:**
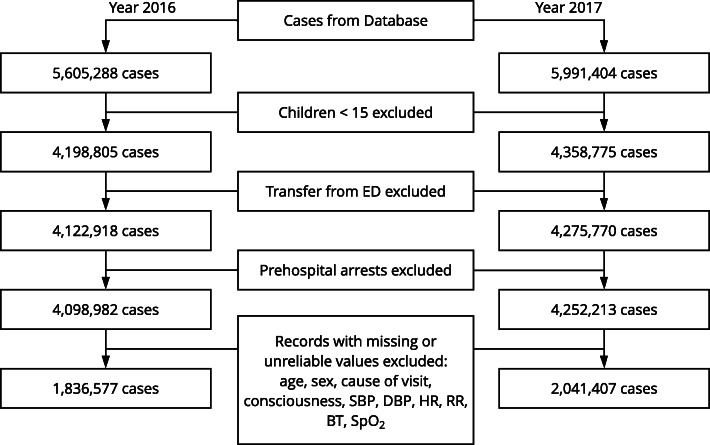
Flow diagram of included and excluded cases analyzed in the present study. Data from 2016 were used for model construction, and data from 2017 were used for validation. ED: emergency department; SBP: systolic blood pressure; DBP: diastolic blood pressure; HR: heart rate; RR: respiratory rate; BT: body temperature; SpO2: pulse oximetry

A total of 5,605,288 cases were extracted from the 2016 database. After applying the exclusion criteria, 1,836,577 cases were finally included for model building. From the 2017 database, 5,991,404 cases were extracted, and 2,041,407 cases were included for further analysis (Fig. 2). The general characteristics of the study population are summarized in Table 1. *P*-values are not presented because the study was not intended to generate comparisons between the years.

**Table 1:** General characteristics of the study population

Year	2016 (*N* = 1,836,577)	2017 (*N* = 2,041,407)
Male	931,366 (50.7%)	1,034,833 (50.7%)
Age	54.0 [36.0–69.0]	55.0 [37.0–70.0]
Event		
Disease	1,451,945 (79.1%)	1,609,997 (78.9%)
Other than disease	384,632 (20.9%)	431,410 (21.1%)
Consciousness		
Alert	1,737,235 (94.6%)	1,933,837 (94.7%)
Responsive to verbal stimuli	54,659 (3.0%)	59,301 (2.9%)
Responsive to pain	38,462 (2.1%)	41,912 (2.1%)
Unresponsive	6221 (0.3%)	6357 (0.3%)
Vital signs		
Systolic BP (mmHg)	130.0 [115.0–147.0]	130.0 [116.0–150.0]
Diastolic BP (mmHg)	80.0 [70.0–90.0]	80.0 [70.0–90.0]
Heart rate (/min)	84.0 [74.0–98.0]	84.0 [74.0–98.0]
Respiratory rate (/min)	20.0 [18.0–20.0]	20.0 [18.0–20.0]
Body temperature (°C)	36.6 [36.4–37.0]	36.6 [36.4–37.0]
Pulse oxygen saturation (%)	98.0 [97.0–99.0]	98.0 [97.0–99.0]
Admission	603,621 (32.9%)	669,544 (32.8%)
ED mortality	5236 (0.3%)	5276 (0.3%)
Mortality after admission	38,568 (6.4%)	42,433 (6.3%)
Overall mortality	43,804 (2.4%)	47,709 (2.3%)

### EDIES scoring system

**Fig. 3:**
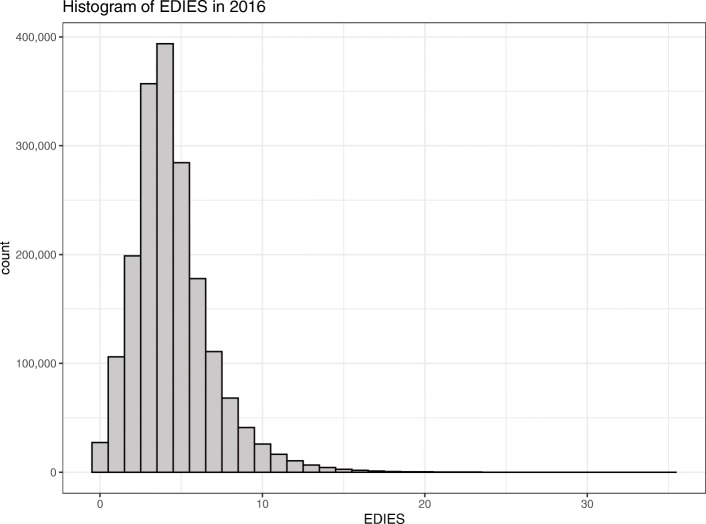
Histogram displaying the distribution of the Emergency Department Initial Evaluation Score (EDIES) within the construction dataset

The results of logistic regression of the multivariate association of mortality and variable scores based on univariate mortality and epidemiologic parameters are summarized in Table 2. The final scoring system is presented in Table 3. Body temperature was removed from the final model because the positive univariate association with mortality was inversed in the multivariate regression. Diastolic blood pressure was removed because of multicollinearity with systolic blood pressure. EDIES can theoretically have values between 0 and 39, although with the 2016 dataset, the EDIES were distributed between 0 and 35. (Fig. 3).

**Table 2:** Multivariate association of epidemiological and physiological parameters for the prediction of mortality. Scores for physiological parameters were arbitrarily allocated based on the univariate association with mortality

Parameter	Coefficient	Standard error	P-value
(intercept)	−6.703	0.022	< 0.001
Male	0.455	0.011	< 0.001
Event is disease	0.786	0.020	< 0.001
AVPU score	0.089	0.001	< 0.001
Age score	0.211	0.002	< 0.001
Heart rate score	0.110	0.001	< 0.001
Respiratory rate score	0.051	0.001	< 0.001
Systolic BP score	0.082	0.001	< 0.001
SpO_2_ score	0.075	0.001	< 0.001

*AVPU* alert, verbal, painful, unresponsive; *BP* blood pressure; *SpO*_*2*_ pulse oxygen saturation

**Table 3:** The Emergency Department Initial Evaluation Score system developed in the present study

Item	Score	Item	Score
Sex		Event	
Male	1	Disease	2
Female	0	Other than disease	0
Age, years		Consciousness (AVPU Scale)	
≥ 15 and ≤ 39	0	Alert	0
≤ 70	1	Respond to verbal stimuli	2
≤ 78	2	Response to pain	3
≤ 85	3	Unresponsive	7
≤ 93	4		
> 93	5		
Systolic blood pressure, mmHg		Heart rate, beats/min	
≤ 21	11	≤ 42	2
≤ 26	10	≤ 54	1
≤ 33	9	≤ 87	0
≤ 39	8	≤ 120	1
≤ 46	7	≤ 140	2
≤ 52	6	> 140	3
≤ 60	5		
≤ 67	4		
≤ 78	3		
≤ 90	2		
≤ 106	1		
≤ 202	0		
> 202	1		
Respiratory rate, breaths/min		Pulse oxygen saturation, %	
≤ 5	6	≤ 82	4
≤ 7	3	≤ 86	3
≤ 13	1	≤ 91	2
≤ 20	0	≤ 96	1
≤ 33	1	> 96	0
> 35	2		

*AVPU* alert, verbal, painful, unresponsive

### Validation of EDIES with the 2017 dataset

**Fig. 4:**
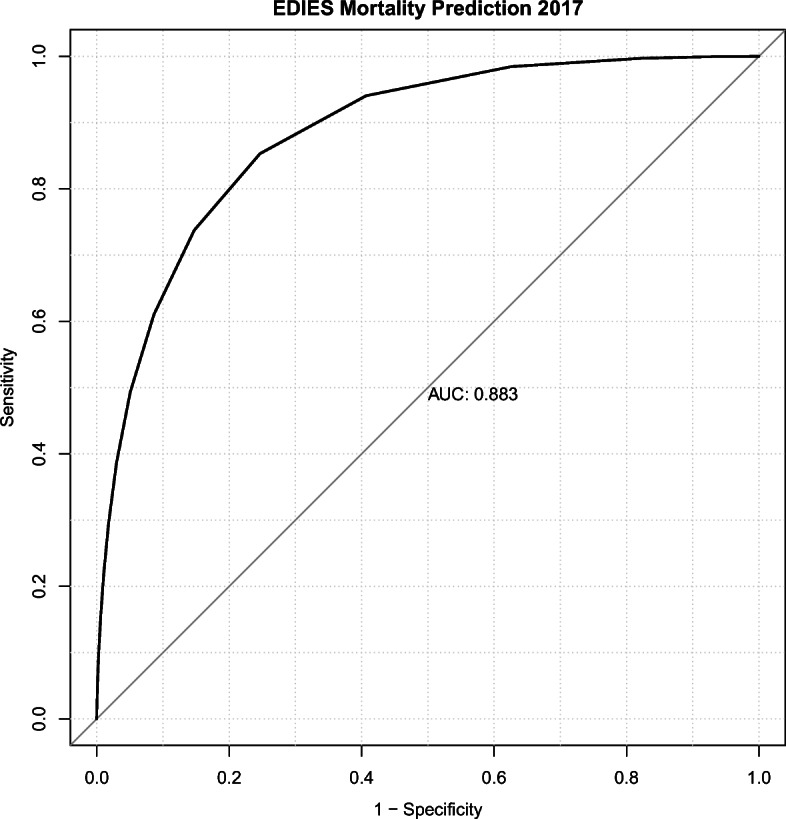
Receiver operating characteristic (ROC) curve presenting the performance of the Emergency Department Initial Evaluation Score (EDIES) for the prediction of mortality. The area under the ROC curve was 0.883 (95% confidence interval: 0.882–0.884)

When EDIES was applied to the dataset from the 2017 NEDIS registry, the area under the receiver operating characteristic curve for the prediction of mortality was 0.883 (95% confidence interval [CI]: 0.882–0.884, Fig. 4).

### Ws statistics

To calculate the Ws statistic, the probability of survival predicted by the scoring system and the fraction for each severity group was derived from the 2016 dataset and are presented in Tables 4 and 5.

**Table 4:** The predicted probability of survival based on the Emergency Department Initial Evaluation Score developed in the present study, based on the 2016 dataset

Score	Predicted probability of survival
0	1.00000
1	0.99973
2	0.99935
3	0.99842
4	0.99511
5	0.98673
6	0.97252
7	0.95153
8	0.92339
9	0.88886
10	0.84543
11	0.79589
12	0.75019
13	0.69430
14	0.95177
15	0.59030
16	0.52206
17	0.49335
18	0.46640
19	0.44246
20	0.40063
21	0.37624
22 and above	0.24753

**Table 5:** The number and fraction of cases from the 2016 dataset for each Emergency Department Initial Evaluation Score value. Used for reference fraction to standardize the severity distribution

Score	Number (fraction)
0	27,248 (0.0148)
1	106,039 (0.0577)
2	198,739 (0.1082)
3	356,919 (0.1943)
4	393,640 (0.2143)
5	284,345 (0.1548)
6	177,881 (0.0969)
7	110,766 (0.0603)
8	68,128 (0.0371)
9	41,147 (0.0224)
10	25,930 (0.0141)
11	16,462 (0.0090)
12	10,616 (0.0058)
13	6670 (0.0036)
14	4319 (0.0024)
15	2763 (0.0015)
16	1768 (0.0010)
17–18	1871 (0.0010)
19 and above	1326 (0.0007)
Total	1,836,577 (1.0000)

The Ws statistic calculated from the 2016 dataset was 0.000 (95% CI: − 0.021 – 0.021). The Ws statistic calculated from the 2017 dataset was 0.049 (95% CI: 0.030–0.069). From the 2017 dataset, 30,000 cases were sampled repeatedly 1000 times using the random, hypothetical nonsevere, and hypothetical severe categories, and the Ws statistic for each distribution was calculated. The mean Ws statistic from 1000 random selections was 0.049, while those from nonsevere selections and severe selections were 0.059 and 0.048, respectively. The chances that the 95% CI of the Ws statistic from 30,000 samples included the population Ws statistic, which was 0.049, were 94.5% for random selection, 96.6% for nonsevere samples, and 96.9% for severe samples.

## Discussion

We developed a severity score for the prediction of survival or mortality from data collected during the initial ED evaluation. Although the predicted probabilities of survival might not be useful as prognostic indicators for individual patients, they allow comparisons of the performance of an institution with the original prediction database or with other institutions [14]. Prediction systems such as the Revised Trauma Score (RTS), the Abbreviated Injury Scale (AIS), the Injury Severity Score (ISS), and the TRISS have been used for prognostication for trauma victims and comparisons of performance between trauma systems for decades [6, 7, 13, 14, 23]. The area under the ROC curve (AUROC) is commonly used to measure the predictive performance of a scoring system, and the AUROCs of trauma scores including the TRISS are reported to be between 0.57 and 0.98 in the literature [7, 23]. There have also been efforts to develop or apply severity scores to general ED patients. Although scoring systems such as the Acute Physiology and Chronic Health Evaluation (APACHE) score or Simplified Acute Physiology Score were developed to predict in-hospital mortality, the application of these systems in the general ED population is limited because they rely on laboratory values that are often not measured in ED patients with relatively mild complaints and are not included in national databases [24–27]. A recent multinational study found that the National Early Warning Score (NEWS) can predict mortality among adult medical patients in the ED with an AUROC of 0.73, and the combination of the NEWS and laboratory biomarkers yielded an AUROC of 0.82 [16]. Some investigators developed machine-learning models for the prediction of death or admission to intensive care units among ED patients and reported the AUROCs of their models to be between 0.84 and 0.87 [15, 28]. The AUROC of the scoring system for survival prediction among general ED patients developed in this study was 0.883, which can be regarded as adequate, considering the fact that patients with diverse complaints were included, no laboratory data are required, and the calculation is much simpler than the calculations needed for trauma scores based on anatomical injuries or sophisticated machine-learning-based models.

W statistics were introduced to compare the clinical performance of trauma care between institutions or trauma systems, and the W statistic represents the average increase in the number of survivors per 100 patients treated compared with reference expectations [14]. Although the W statistics are being utilized to compare treatment outcomes among trauma care systems, they have been criticized as inappropriate for comparing systems with different case severity distributions [10–13]. To address the severity distribution issue, the Ws statistic was proposed as an alternative. The Ws statistic is calculated by standardizing the W statistic with respect to injury severity ranges, in a similar manner to calculating age-standardized mortality rates. The range of severities should be divided into a number of intervals, and the W statistic of each interval is calculated and multiplied by the fraction in the reference population and then summarized into the Ws statistic [13].

Although the W and Ws statistics are based on the TRISS, which was developed for use in trauma settings, the principle underlying the W statistic could be applied to other areas of patient care if appropriate scoring systems are used instead of the TRISS. We developed the EDIES and calculated the Ws statistic based on the EDIES instead of the TRISS, and validated its use for comparing severity-adjusted mortality between institutions with different severity distributions. As the intervals of severity were defined by the authors and the interval definition would affect the validity of the Ws statistic in populations with different case severity distributions, the Ws statistic was validated by taking samples with different severity distributions and comparing the sample Ws statistic with the population Ws statistic. The sample Ws statistics were found to be similar to the population Ws, although the severity distributions of the hypothetical samples were extremely different from that of the population. To assess the statistically significant difference between Ws statistics, the 95% confidence intervals (CI) of the Ws statistic were used [13]. To assess whether the Ws statistics in different severity samples and their calculated 95% CIs can represent the population Ws statistic, samples were taken 1000 times from each severity classification, and the number in which the 95% CI for the sample Ws statistic included the population Ws statistic was counted. Theoretically, it is expected that the population Ws statistic is within the 95% CIs of the sample Ws statistics in 95% of cases, and the results showed that the chances were between 94.5 and 96.9%. Therefore, the Ws statistic and its 95% CI are stable, even with very different severity distributions.

### Limitations

While the data were collected from all emergency centers nationally, data from small community EDs were not included; therefore, application of the system to those EDs might be inappropriate. The relatively high rate of exclusion because of missing values may have caused selection bias; more cases were likely included from centers with relatively high proportions of patients with severe cases, which were also likely to have policies in place regarding the recording of vital signs and detailed medical records. Although some existing scoring systems have been derived from populations with fewer missing values, they tend to have been derived from the data obtained in a single hospital, including the Rapid Emergency Medicine Score (REMS) and ViEWS, which later served as a template for NEWS [15, 29–31]. Our results are more reliable than the results in those studies because our data were derived from EDs at various levels and of different sizes; however, the amount of missing data was a limitation.

## Conclusions

In conclusion, the scoring system developed in the present study utilizing the parameters gathered during the initial ED evaluation has acceptable performance for the prediction of in-hospital mortality. The Ws statistics based on the scoring system can be used to compare the performance of an ED with the reference data or with the performance of other institutions.

## Data Availability

The data that support the findings of this study are available from the National Emergency Medical Center of the Republic of Korea, but restrictions apply to the availability of these data, which were used under license for the current study and thus are not publicly available.

## Abbreviations

ED: Emergency Department
TRISS: Trauma and Injury Severity Score
NEDIS: National Emergency Department Information System
AVPU: Alert, verbal, pain, unresponsive
EDIES: Emergency Department Initial Evaluation Score
ROC: Receiver operating characteristic
Ws: Standardized W
RTS: Revised Trauma Score
AIS: Abbreviated Injury Scale
ISS: Injury Severity Score
AUROC: Area under the receiver operating characteristic curve
APACHE: Acute Physiology and Chronic Health Evaluation
NEWS: National Early Warning Score
REMS: Rapid Emergency Medicine Score
